# Diseases of the Lungs, Heart and Kidney

**Published:** 1893-07

**Authors:** 


DISEASES OF THE LUNGS, HEART AND KIDNEYS, by N. S. Davis, Jr., A.M., M. D., Professor Principles and Practice of Medicine, Chicago Medical College; Physician to Mercy Hospital; Member American Medical Association, Illinois State Medical Society. Philadelphia and London; The F. A. Davis Co. 1892.

This is No. 14 in the Physicians' and Students' Ready Reference Series, and comprises portions of lectures delivered by the author in the Chicago Medical College. The book will be of value to students and the young physician. Much space is devoted to *treatment*, and the indications for and mode of action of drugs in each disease.

